# The complete mitochondrial genome of the giant casemaker caddisfly *Phryganea cinerea* (Insecta: Trichoptera: Phryganeidae)

**DOI:** 10.1080/23802359.2018.1450686

**Published:** 2018-03-16

**Authors:** Living Prairie Mitogenomics Consortium

**Affiliations:** Department of Biological Sciences, University of Manitoba, Winnipeg, MB, Canada

**Keywords:** Illumina sequencing, mitogenomics, inquiry-based learning, Trichoptera, Phryganeidae

## Abstract

The rush sedge caddisfly *Phryganea cinerea* Walker, 1852 (Phryganeidae, the giant casemakers), is a widespread and adaptable North American caddisfly. Genome skimming by Illumina sequencing permitted the assembly of a complete 15,043 bp circular mitogenome from *P. cinerea* consisting of 78.2% AT nucleotides, 22 tRNAs, 13 protein-coding genes, 2 rRNAs and a control region in the ancestral insect gene order. *Phryganea cinerea COX1* features an atypical CGA start codon and *COX1, NAD1, NAD4,* and *NAD5* exhibit incomplete stop codons completed by the addition of 3′ A residues to the mRNA. Phylogenetic reconstruction reveals a monophyletic Order Trichoptera and Family Phyrganeidae.

The Living Prairie Mitogenomics Consortium is a project to construct a library of arthropod mitogenomes for improved DNA-based species identification and phylogenetics (Living Prairie Mitogenomics Consortium 2017; Marcus 2018). Mitogenome sequences were annotated by undergraduates in a course inquiry-based learning exercise (Marcus et al. 2010). Students who analyzed the data successfully (which were further curated by the instructor) belonged to our consortium.

On 30–31 July 2015, a USDA blacklight trap (Winter 2000) was deployed to collect night-flying insects at the Living Prairie Museum (LPM, GPS 49.889607 N, -97.270487 W). Nearby aquatic habitats include Sturgeon Creek (0.57 km) and the Assiniboine River (1.92 km) (Marcus 2018). One adult specimen of the rush sedge caddisfly *Phryganea cinerea* Walker, 1852 (Insecta: Trichoptera: Phryganeidae, the giant casemakers, project specimen number 2015.07.30.014) was trapped, pinned and deposited in the Wallis Roughley Museum of Entomology at the University of Manitoba (voucher JBWM0360830).

*Phryganea cinerea* larvae are common in North American marshes and littoral zones of lakes (Williams and Penak 1980), but are also found at depths of up to 100 m (Selgeby 1974) and in sluggish streams (Hilsenhoff 1970). Larvae preferentially make cases from shredded vegetation, but will use many other materials (Neave 1933; Williams and Penak 1980). To lay eggs, adult female *P. cinerea* will fly up to 10 m in the air and dive-bomb the water surface, sending up small splashes, mimicking rain (LaFontaine 1981). Presented here is the first complete New World mitogenome for family Phryganeidae from *P. cinerea.*

DNA was prepared (McCullagh and Marcus 2015) and sequenced by Illumina MiSeq (San Diego, CA) (Peters and Marcus 2017). The mitogenome of *P. cinerea* (Genbank MG980616) was assembled by Geneious 10.1.2 from 6,093,858 paired 75 bp reads using a *Eubasilissa regina* (Trichoptera: Phryganeidae) reference mitogenome (NC023374) (Wang et al. 2014). Annotation was in reference to *E. regina* and *Anabolia bimaculata* (Trichoptera: Limnephilidae, MF680449) mitogenomes (Peirson and Marcus 2017). The *T. tardus* nuclear rRNA repeat (Genbank MG986214) was also assembled and annotated using *A. bimaculata* (MF680448) and *Triaenodes tardus* (Trichoptera: Leptoceridae, MG201853) (Lalonde and Marcus 2017) reference sequences.

The *P. cinerea* circular 15,043 bp mitogenome assembly was composed of 19,655 paired reads with nucleotide composition: 39.2% A, 14.0% C, 7.7% G, and 39.0% T. Gene composition and order in *P. cinerea* is identical to most other trichopteran mitogenomes (Marcus 2018). *Phryganea cinerea COX1* begins with an aberrant start codon (CGA) that is typical of insects (Liao et al. 2010). The mitogenome contains four protein-coding genes (*COX1, NAD1, NAD4, NAD5*) with single-nucleotide (T) stop codons completed by post-transcriptional addition of 3′ A residues. The tRNAs, rRNAs, and control region are typical for Trichoptera (Lalonde and Marcus 2017; Peirson and Marcus 2017).

The mitogenomes from *P. cinerea,* 14 other Trichoptera, and 6 species from sister order Lepidoptera were aligned in CLUSTAL Omega (Sievers et al. 2011) and analyzed by maximum likelihood (ML) and parsimony in PAUP* 4.0b8/4.0d78 (Swofford 2002) (Figure 1). Phylogenetic analysis reveals a monophyletic Trichoptera and places *P. cinerea* as sister to *E. regina* in monophyletic family Phryganeidae.

**Figure 1.**
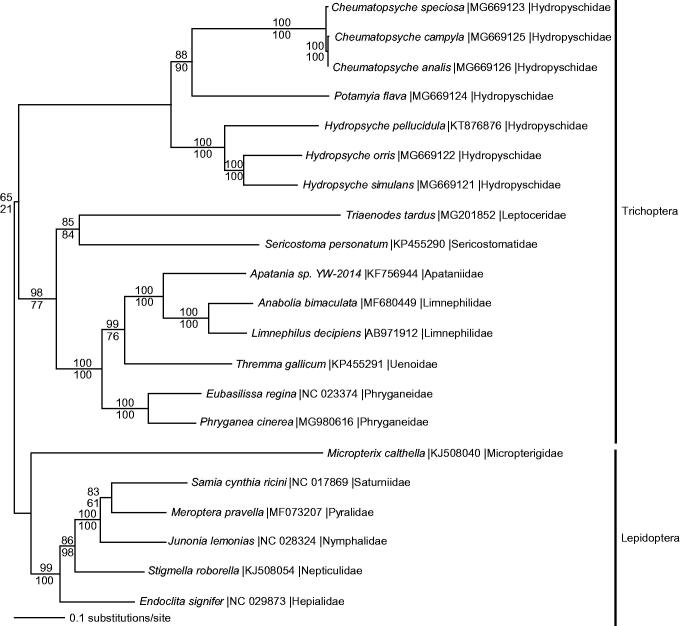
Maximum likelihood phylogeny of superorder Amphiesmenoptera (GTR + I + G model, I = 0.1730, G = 0.9090, likelihood score 186552.81293) included complete mitochondrial genome sequences from *Phryganea cinerea*, 14 other Trichoptera species, and 6 representatives from sister clade Lepidoptera based on 1 million random addition heuristic search replicates (with tree bisection and reconnection). One million maximum parsimony heuristic search replicates also produced a single tree (40,342 steps) with a topology identical to the ML tree. Maximum likelihood bootstrap values are above nodes and maximum parsimony bootstrap values are below nodes (each from 1 million random fast addition search replicates).
